# Food and Nutrient Displacement by Walnut Supplementation in a Randomized Crossover Study

**DOI:** 10.3390/nu14051017

**Published:** 2022-02-28

**Authors:** Zuhair S. Natto, Gina Siapco, Karen Jaceldo-Siegl, Ella H. Haddad, Joan Sabaté

**Affiliations:** 1 Department of Dental Public Health, Faculty of Dentistry, King Abdulaziz University, Jeddah 21589, Saudi Arabia; znatto@kau.edu.sa; 2 Center for Nutrition, Healthy Lifestyle & Disease Prevention, School of Public Health, Loma Linda University, Loma Linda, CA 92350, USA; gsiapco@llu.edu (G.S.); ehaddad@llu.edu (E.H.H.); jsabate@llu.edu (J.S.); 3 School of Osteopathic Medicine, University of the Incarnate Word, San Antonio, TX 78235, USA

**Keywords:** food displacement, nutrient displacement, walnut, crossover

## Abstract

The aim of this article is to evaluate the effect of a daily supplement of walnuts on the overall daily diet and nutrient profile of healthy adults. A randomized controlled trial with crossover design was conducted for two 6-month diet periods in southeast Californian communities. Subjects were randomized to receive a control diet or a walnut-supplemented diet, then switched. The walnut supplement represented approximately 12% of their daily energy intake. Trained nutritionists collected seven 24 h dietary recalls from each participant (a total of 14 recalls for both periods). Ninety participants were able to complete the study, including 50 females and 40 males. The average age of the participants was 54.3 years. Diets in the walnut period had significantly higher vegetable protein, total fat, total PUFA, PUFA 18:2, PUFA 22:6, and total dietary fiber (*p* < 0.05), while also exhibiting significantly lower PUFA 20:5. All mineral levels were higher on the walnut-supplemented diet. Calcium, phosphorus, magnesium, and zinc were, particularly, significantly higher among the walnut-supplemented group (*p* < 0.05). Displacement occurred in more than one-third of the entire nuts and seeds group; four-fifths of the non-alcoholic beverages and desserts groups; and the majority of the candy, sugar, and sweets group. Walnut supplementation can lead to favorable modifications in nutrient and food intake profiles that may contribute to chronic disease prevention. Nutrient and food displacement may be a mechanism to explain the favourable association between walnut intake and improved diet.

## 1. Introduction

A strong association between daily dietary patterns and health status is recognized by the World Health Organization and several other nutrition councils and boards worldwide [1,2], and is addressed in the guidelines released by these organizations regarding the consumption of foods that promote health. One of these foods is walnut, whose cardio-protective effect on health is supported by substantial evidence [3,4,5]. Walnut can also increase the dietary intake of certain beneficial nutrients associated with a lower risk of developing type 2 diabetes mellitus risk factors [6,7]. The US Food and Drug Administration (FDA), the American Health Association (AHA), and the Academy of Nutrition and Dietetics (AND), therefore, recommend a daily walnut intake of 1.5 oz (42 g) [2,8].

Several large epidemiological studies have proven the protective effects of nut consumption on cardiovascular health [9,10,11] and blood lipid profiles [5,12,13,14,15], and in preventing cardiovascular disease complications and early death among individuals with type 2 diabetes [16,17]. Although considered a dense source of energy due to their fat content, regular nut consumption has not been implicated in long-term weight gain. A six-month study on almond supplementation concluded that while it may have led to a small amount of weight gain in the short term [18], it did not change body weight, % body fat, waist circumference, nor waist-to-hip ratio, after two years of follow-up. In our previous investigation, walnuts accounting for 12% of energy intake, added daily to the habitual intake of free-living subjects, did not significantly affect body weight and body composition after adjusting for energy intake [19]. These results were confirmed by a systematic review and meta-analysis of 33 clinical studies, which found that increased nut consumption did not increase adiposity [20].

Nuts are known for their favorable fatty acid profiles, mainly monounsaturated and polyunsaturated fats that provide about 80% of fat calories, and significant quantities of minerals and vitamins known for their antioxidant properties, such as beta-carotenes, vitamin E, and selenium [4]. Walnuts, in particular, are rich in alpha-linolenic acid, the essential n-3 fatty acid, which is a precursor to the long-chain n-3 fatty acids. The incorporation of nuts in the diet could favorably improve nutrient intake [21,22]. Thus, in the current research, we have evaluated the effect of a daily walnut supplement on the overall daily diet and nutrient profile of healthy subjects.

## 2. Materials and Methods

Details of the methods for this study were published earlier [19]. The methods are briefly described here, and the CONSORT guidelines were followed.

### 2.1. Subjects

Participants were Southeast California community residents that met the following inclusion criteria: less than 1 kg of body weight change in the last six months, BMI < 35 kg/m^2^, and nut intake less than once a week. Individuals with a diagnosed metabolic disorder that might affect body weight or who have a known nut allergy were excluded. Based on the original intent of this study, a sample size of 80 participants would suffice to detect a 0.5 kg weight change at α-level of 5% and 80% power. A total of 94 individuals enrolled in the study but 2 had compliance difficulty and 2 were diagnosed with a metabolic disorder while in the study so they were withdrawn.

### 2.2. Study Design

The study was a free-living randomized controlled trial with a crossover design comprising two 6-month diet periods. Participants were randomized to either a walnut-supplemented-to-control diet or a control-to-walnut-supplemented diet. Clinics were held at baseline and every two months afterwards during which time, anthropometric measurements and blood samples were taken. Anthropometric measurements were taken at baseline and at every clinic.

### 2.3. Dietary Interventions

Participants were instructed to eat their usual or habitual diet while in the study. During the 6-month walnut-supplemented phase, participants were provided their daily allotment of walnuts which accounted for approximately 12% of their daily energy intake. No dietary advice was given except that during the control diet phase, participants were instructed to avoid eating walnuts and/or advised to consume no more than 1 serving per week of other nuts. Participants were further instructed not to change their physical activity levels or try to lose weight while in the study.

The amount of walnuts provided was initially based on energy expenditure calculations from the WHO equations [23] then modified based on the daily energy intake observed in the 24 h dietary recalls. The individualized walnut packs were labeled with the name of the participant, the required amount per day and day of the week; these, together with additional bags for family members, were provided free of charge. Individuals received their portion every two months when they came for the clinic visits. Participants were also asked to return any unconsumed walnuts. To check participants’ compliance, erythrocyte membrane concentration of fatty acid was measured at the end of each six-month period as a biological marker of their adherence to the intervention.

### 2.4. Measurement of Dietary Intake

Trained nutritionists collected 24 h dietary recalls from participants through unannounced and non-consecutive telephone interviews. Participants provided seven recalls for each diet phase, a total of 14 recalls for both phases. The recall consisted of two weekend days and five weekdays to capture all possible daily variations in intake.

### 2.5. Data Analysis

#### 2.5.1. Estimation of Nutrient Displacement

The estimated nutrient displacement (D*_i_*) was calculated as described in previous works [18,21,24]. In this study, the control diet represents the habitual diet (H), and the walnut-supplemented diet (W) is a modified habitual diet which incorporates the walnut supplement (S). Because the walnut supplement was added to the habitual diet, we can calculate the expected intake as the sum of the habitual diet and the walnut supplement (H + S), which would be the expectation if no displacement occurs. To estimate nutrient displacement (D), we subtracted the observed intake of a nutrient *i* in the walnut-supplemented diet from the expected intake of that nutrient as in the following formula: D*_i_* = (H*_i_* + S*_i_*) − W*_i_*. Percentage of nutrient displacement was estimated by D*_i_*/S*_i_* × 100, which is an inverse measure of the degree to which the walnut supplement induces a change in the intake of a specific nutrient [18,21,24]. Different levels of nutrient displacement that occur in the walnut-supplemented diet may be interpreted as follows: (1) no displacement (0%) means that the net effect of the walnut supplement is the addition of a specific walnut nutrient to the total intake of that nutrient, (2) partial displacement (more than zero and less than 100%) means that a specific nutrient in the walnut supplement reduced the amount of that nutrient from non-walnut food sources, and the net effect of the walnut supplement is an increase in the intake of that nutrient, (3) full displacement (100%) means that a nutrient in the walnut supplement reduced an equal amount of that nutrient from other foods, and the net effect is zero change in the intake of that nutrient, (4) more than fully displaced (>100%) means that a specific nutrient in the walnut supplement replaced what little amount of that nutrient is contained in non-walnut food sources, and the net effect is that the walnut-supplemented diet contains less of that nutrient, (5) negative displacement (any percentage with a “–” sign) occurs when the walnut-supplemented diet contains more of a specific nutrient than the habitual or control diet, thus the negative net effect. In this condition, the walnut-supplemented diet retains that nutrients from both non-walnut and walnut sources. We applied a similar approach to estimate food displacement.

#### 2.5.2. Statistical Analysis

The mean and standard deviations of the seven diet recalls were calculated. After a normality check, a paired *t*-test was used to compare intakes during the habitual and walnut-supplemented diet periods, and independent sample *t* test to compare between males and females. Displacement of any food or nutrient was calculated by subtracting the observed intake of that nutrient/food in the supplemented diet from the expected intake of that nutrient/food, after which the percentage of displacement was calculated accordingly. All analyses were performed using SAS statistical analysis software package, version 9.3 (SAS Institute Inc., Cary, NC, USA). A *p* value < 0.05 was considered as statistically significant.

## 3. Results

### 3.1. Subjects

Ninety participants were able to complete the study, including 50 women and 40 men. The mean (SD) age of the participants was 54.3 (10.6) years. Half of the participants were overweight (52.8%), followed by normal weight (33.0%) and obese (14.2%). Table 1 shows the baseline characteristics of these participants.

### 3.2. Nutrient and Food Profile

Data for male and female participants were reported separately due to the potential differences between their intakes (Table 2 and Table 3). None of the results were statistically significant between males and females, except for PUFA 18:2 (*p* = 0.0252), PUFA 18:3 (*p* = 0.0001), and alcohol (*p* = 0.0346). Overall, males had a higher intake of macronutrients, vitamins, and minerals compared with females. Table 2 presents the changes in intake of selected macro- and micronutrients in both groups. Mean total energy intake increased significantly (*p* < 0.01) during the walnut-supplemented diet compared with the habitual diet. On average, intake during the walnut period contained significantly higher vegetable protein, total fat, total PUFA, linoleic acid (PUFA 18:2), α-linolenic acid (PUFA 18:3), and total dietary fiber (*p* < 0.05), and significantly lower eicosatetraenoic acid (PUFA 20:5) and docosahexaenoic acid (PUFA 22:6).

The intake of vitamins was higher or relatively similar in the walnut-supplemented diet compared with the habitual diet. However, none were statistically significant (Table 3). The intake of most minerals was higher during the walnut-supplemented diet, particularly calcium, phosphorus, magnesium, and zinc (*p* < 0.05) (Table 3).

### 3.3. Nutrient and Food Displacement

Table 4, Table 5 and Table 6 illustrate the estimated displacement percentages of macronutrients, micronutrients, and food after walnut supplementation.

In the walnut-supplemented diet, most of the foods were more than fully displaced (Table 4). The estimated displacements in the walnut-supplemented diet for the grains, breads, and starches group; the vegetables and legumes group; the fruits and related products group; the meat, fish, and poultry group, were 128, 112, 104, and 116 percent, respectively. More than one-third of the nuts and seeds group; four-fifths of the non-alcoholic beverages and desserts groups; the majority of the candy, sugar, and sweets group were displaced.

In the walnut group, approximately one-half of the total energy, two-thirds of the total dietary fiber, 80% of the total protein, and 308% of the total carbohydrate present in walnuts were displaced (Table 5). Displaced dietary fatty acids comprised 58% of total fat, 76% of SFA, 77% of MUFA, and 57% of PUFA in the walnut-supplemented diet. The estimated displacement in the walnut group for carbohydrate and sugars was 252%.

Displacement estimates for total vitamin A, total folate, beta-carotene, retinol, Ca, P, Mg, Fe, Zn, Cu, and K ranged from 48 to 64% in the walnut-supplemented diet. The micronutrients with displacement estimates >100% were vitamin C and total vitamin E. 

## 4. Discussion

Our results show that a daily intake of walnuts (12% of total energy intake) can induce favorable nutrient modifications to an individual’s habitual diet. A significant increase was observed in the intake of total PUFA, linoleic acid (PUFA 18:2), alpha-linolenic acid (PUFA 18:3), vegetable protein, total fat, total dietary fiber, calcium, phosphorus, magnesium, and zinc, in addition to a significant reduction in eicosatetraenoic acid or EPA (PUFA 20:5) and docosahexaenoic acid or DHA (PUFA 22:6). Intake levels of these nutrients largely met the daily reference intake and the dietary recommendations to prevent chronic diseases and improve cardiovascular health. The significant increase in total fat intake in the walnut group could be due to a higher intake of total, 18:2, and 18:3 PUFA, which might reduce the risk of coronary heart disease.

Moreover, animal protein had been more than completely displaced by protein from walnuts and this is despite the lack of dietary advice regarding protein intake during the course of the study. The total energy obtained from carbohydrate was higher in the control diet than in the walnut diet. However, total protein and total fat were higher in the walnut diet, although only the total fat significantly differed, possibly due to the walnut intake.

Walnut supplementation increased the intake of most minerals and vitamins, such as total vitamin A, total folate, beta-carotene, retinol, calcium, phosphorus, magnesium, iron, zinc, copper, and potassium. Our findings regarding calcium, phosphorus, magnesium, and zinc levels being significantly higher during the walnut supplementation phase were similar to the findings of Bitok and colleagues [21].

The concept of nutrient displacement has been investigated in only a few walnuts/nuts studies [15,21,24,25], possibly due to the difficulty of assessing changes in dietary patterns among free-living individuals. Although previous studies have shown that diet supplementation with various nuts consistently results in partial displacement of energy, protein, total fat, MUFA, and PUFA [15,21,24,25], considerable variations can be found between these studies. The results of the current study confirm the previous results, documenting at least one-half displacement of the total energy, total dietary fiber, total protein, total carbohydrate, total fat, SFA, MUFA, PUFA, carbohydrate, and sugars. General displacement patterns tend toward a healthier nutrient profile with the nut supplement, which can improve cholesterol, blood pressure, and blood sugar levels.

In this study, non-alcoholic beverages; desserts; candy, sugar, and sweets were partially displaced by walnuts. These types of foods might be associated with weight gain [26,27,28], reduced bone strength [29,30], nutritional deficiencies [29,30], dental caries [31,32], hypertension [3,5,14,33], type 2 diabetes [28,34,35], cardiovascular disease [3,5,12,13,14], metabolic syndrome [27], and non-alcoholic fatty liver disease [36]. Walnuts displaced seven food groups by more than 110%, which means that participants ate less of these food groups in the walnut-supplemented diet than in the habitual diet. These included grains, breads, starches; vegetables and legumes; meat, fish and poultry; fats and oils, nuts (other than walnuts) and seeds; water. This displacement pattern coincided with the displacement of selected nutrients. For example, a lower intake of meat, fish and poultry was consistent with less intake of animal protein and cholesterol. The negative displacement of PUFA 18:2 means that the walnut-supplemented diet had more PUFA 18:2 than would be expected, had this nutrient simply been added to the habitual diet. This is not surprising, since PUFA 18:2 is the most abundant fatty acid in walnuts, and further suggests that the walnut-supplemented diet retained not only the linoleic acid from walnuts but also from non-walnut sources.

The concept of nutrient displacement can be best tested when a comparison of intake can be done on the same individuals. Thus, the present study’s crossover design is a strength, since this allowed us absolute displacement computations. Another strength is the high rate of compliance with the protocol, regarding intake of walnuts during the walnut diet phase, and non-intake of walnuts during the control diet phase [37], which gives credence to the measurement of displacement due to walnut consumption. One drawback of this study is the self-reported dietary assessment method, which can lead to error and under- or over-estimation [38,39]. However, both diet phases were evaluated with the same dietary assessment method, unannounced 24-h diet recalls. In addition, other factors, such as absorption efficiency, should be investigated in future research.

## 5. Conclusions

Walnut intake was able to improve the nutrient and food intake, and partially displace total energy, total dietary fiber, total protein, total carbohydrate, total fat, SFA, MUFA, PUFA, carbohydrate, and sugars. Our findings indicate that supplementing a diet with walnuts can lead to favorable modifications in nutrient intake profiles that may contribute to chronic disease prevention. Thus, nutrient and food displacement may be a mechanism to explain the favorable association between walnut intake and improved diet.

## Figures and Tables

**Table 1:** Baseline characteristics of study participants.

Variable	Mean ± SD	*n* (%)
Age (years)	54.30 ± 10.62	
BMI (kg/m^2^)	26.37 ± 3.58	
Weight (kg)	75.91 ± 14.25	
Height (cm)	148.87 ± 10.37	
Gender		
Male		40 (43.0)
Female		50 (57.0)
BMI classifications		
Normal (18.5–24.9)		30 (33.0)
Overweight (25–29.9)		48 (52.8)
Obese (≥30)		13 (14.2)

**Table 2:** Changes in the intake of selected macronutrients of 90 participants evaluated by seven 24 h recalls for each diet period.

Variable	Male			Female			All		
	Walnut-Suppl Diet (W)	Habitual Diet (H)	Difference (W − H)	Walnut-Suppl Diet (W)	Habitual Diet (H)	Difference (W − H)	Walnut-Suppl Diet (W)	Habitual Diet (H)	Difference (W − H)
	Mean ± SD	Mean ± SD	Mean ± SD	Mean ± SD	Mean ± SD	Mean ± SD	Mean ± SD	Mean ± SD	Mean ± SD
Macronutrients									
Energy, kcal	2274.6 ± 502.0	2186.6 ± 604.9	87.9 ± 368.3	1638.5 ± 358.2	1511.3 ± 343.2	127.2 ± 211.7 *	1914.1 ± 529.3	1804.0 ± 579.4	110.2 ± 288.9 *
Total Carbohydrate, g	286 ± 75.6	295.4 ± 82.9	−9.4 ± 50.1	201.4 ± 52.3	202.5 ± 56.5	−1.2 ± 35.3	238.0 ± 75.8	242.8 ± 82.9	−4.8 ± 42.3
Total Protein, g	81.8 ± 20.8	86.16 ± 32.47	−0.86 ± 19.6	61.4 ± 14.5	59.7 ± 14.6	1.7 ± 10.5	70.3 ± 20.2	69.7 ± 22.4	0.6 ± 15.1
Animal Protein, g	48.0 ± 18.9	49.7 ± 20.7	−1.7 ± 17.7	36.0 ± 12.2	37.9 ± 13.3	−1.9 ± 10.4	41.2 ± 16.5	43.0 ± 17.8	−1.8 ± 13.9
Vegetable Protein, g	33.3 ± 8.5	32.5 ± 13.0	0.8 ± 8.6	25.0 ± 6.1	21.4 ± 7.0	3.6 ± 6.3 *	28.6 ± 8.3	26.2 ± 11.4	2.4 ± 7.5 *
Total Fat, g	93.2 ± 25.6	77.5 ± 28.2	15.7 ± 19.8 *	69.5 ± 18.7	52.9 ± 16.6	16.6 ± 10.8 *	79.8 ± 24.8	63.5 ± 25.4	16.2 ± 15.3 *
Total SFA, g	24.8 ± 8.6	25.2 ± 10.1	−0.4 ± 7.8	18.8 ± 7.1	17.4 ± 6.5	1.4 ± 4.1 *	21.4 ± 8.3	20.8 ± 9.1	0.6 ± 6.0
Total MUFA, g	29.1 ± 10.0	29.6 ± 11.1	−0.5 ± 8.6	21.0 ± 6.7	19.9 ± 6.5	1.1 ± 4.7	24.5 ± 9.2	24.1 ± 10.0	0.4 ± 6.7
MUFA 18:1, g	27.4 ± 9.4	27.7 ± 10.4	−0.4 ± 8.1	19.7 ± 6.4	18.6 ± 6.1	1.1 ± 4.5	23.0 ± 8.7	22.6 ± 9.4	0.5 ± 6.3
Total PUFA, g	32.4 ± 7.3	16.3 ± 6.5	16.1 ± 5.6 *	24.8 ± 5.2	11.3 ± 4.4	13.5 ± 3.7 *	28.1 ± 7.2	13.5 ± 5.9	14.6 ± 4.8 *
PUFA 18:2. g	27.2 ± 6.3	14.4 ± 5.9	12.8 ± 5.0 *	20.6 ± 4.5	9.8 ± 3.9	10.8 ± 3.3 *	23.5 ± 6.3	11.8 ± 5.4	11.7 ± 4.2 *
PUFA 18:3, g	5.0 ± 1.0	1.5 ± 0.7	3.5 ± 0.8 *	4.0 ± 0.8	1.2 ± 0.5	2.8 ± 0.5 *	4.4 ± 1.0	1.3 ± 0.6	3.1 ± 0.7 *
PUFA 20:4, g	0.1 ± 0.1	0.1 ± 0.1	−0.0 ± 0.1	0.1 ± 0.0	0.1 ± 0.0	−0.0 ± 0.0	0.1 ± 0.1	0.1 ± 0.1	−0.0 ± 0.1
PUFA 20:5, g	0.0 ± 0.0	0.1 ± 0.1	−0.0 ± 0.1 *	0.0 ± 0.0	0.1 ± 0.1	−0.0 ± 0.1	0.0 ± 0.0	0.1 ± 0.1	−0.0 ± 0.1 *
PUFA 22:6, g	0.1 ± 0.1	0.1 ± 0.2	−0.0 ± 0.1 *	0.1 ± 0.1	0.1 ± 0.1	−0.0 ± 0.1	0.1 ± 0.1	0.1 ± 0.1	−0.0 ± 0.1 *
Alcohol, g	4.0 ± 7.9	3.7 ± 7.5	0.3 ± 2.7	1.7 ± 2.7	2.9 ± 5.1	−1.2 ± 3.7	2.7 ± 5.6	3.3 ± 6.2	−0.6 ± 3.4
Cholesterol, mg	241.2 ± 109.1	257.9 ± 144.7	−16.7 ± 127.3	177.1 ± 80.8	196.4 ± 78.6	−19.3 ± 63.7	204.9 ± 98.8	223.1 ± 115.5	−18.2 ± 95.9
Total Dietary Fiber, g	24.2 ± 6.3	23.6 ± 6.8	0.6 ± 3.6	18.8 ± 4.6	16.7 ± 4.3	2.1 ± 4.4 *	21.1 ± 6.0	19.7 ± 6.5	1.5 ± 4.1 *

* *p* value < 0.05.

**Table 3:** Changes in the intake of specific micronutrients of 90 participants evaluated by seven 24 h recalls for each diet period.

Variable	Male			Female			All		
	Walnut-Suppl Diet (W)	Habitual Diet (H)	Difference (W − H)	Walnut-Suppl Diet (W)	Habitual Diet (H)	Difference (W − H)	Walnut-Suppl Diet (W)	Habitual Diet (H)	Difference (W − H)
	Mean ± SD	Mean ± SD	Mean ± SD	Mean ± SD	Mean ± SD	Mean ± SD	Mean ± SD	Mean ± SD	Mean ± SD
Vitamins									
Total Vitamin A, µg	1289.6 ± 804.7	1249.6 ± 672.6	40.0 ± 563.8	1023.9 ± 538.1	954.8 ± 458.2	69.2 ± 406.3	1139.1 ± 675.7	1082.5 ± 576.8	56.5 ± 478.2
Vitamin C, mg	113.2 ± 50.7	106.6 ± 49.8	6.6 ± 257.9	94.0 ± 46.5	100.2 ± 43.8	−6.1 ± 33.2	102.3 ± 49.0	102.9 ± 46.3	−0.6 ± 39.6
Total Folate, µg	451.3 ± 103.2	454.0 ± 143.2	−2.7 ± 102.0	361.7 ± 107.4	348.4 ± 114.8	13.3 ± 98.0	400.5 ± 114.1	394.2 ± 137.6	6.3 ± 99.5
Beta-Carotene, µg	4622.9 ± 4401.4	4376.1 ± 3215.2	246.8 ± 3276	3868.5 ± 2933.8	3497.3 ± 2399.9	371.2 ± 2254	4195.4 ± 3639.8	3878.0 ± 2800.2	317.3 ± 2727.6
Retinol, µg	519.1 ± 469.2	520.2 ± 490.5	−1.1 ± 176.9	379.2 ± 243.0	371.9 ± 265.9	7.3 ± 124	439.8 ± 363.4	436.2 ± 384.6	3.7 ± 148.4
Total Vit E, mg	10.1 ± 3.0	10.9 ± 4.2	−0.7 ± 4.1	8.4 ± 3.9	8.2 ± 4.3	0.2 ± 3.1	9.2 ± 3.6	9.4 ± 4.4	−0.2 ± 3.5
Minerals									
Calcium, mg	855.9 ± 306.7	841.3 ± 313.5	14.6 ± 247.8	732.3 ± 376.8	679.7 ± 348.5	52.6 ± 176.8 *	785.8 ± 351.7	749.7 ± 341.6	36.1 ± 210.1 *
Phosphorus, mg	1394.5 ± 328.6	1352.7 ± 381.0	41.9 ± 273.4	1082.3 ± 338.1	994.8 ± 320.5	87.5 ± 156.7 *	1217.6 ± 366.8	1149.9 ± 389.2	67.7 ± 215.0 *
Magnesium, mg	376.9 ± 77.7	344.3 ± 90.1	32.6 ± 60.4 *	294.5 ± 72.1	255.7 ± 72.2	38.8 ± 44.7 *	330.2 ± 84.8	294.1 ± 91.3	36.1 ± 51.8 *
Iron, mg	17.6 ± 5.3	17.6 ± 5.2	−0.1 ± 5.3	13.2 ± 4.3	12.4 ± 4.6	0.8 ± 3.3	15.1 ± 5.2	14.7 ± 5.5	0.4 ± 4.3
Zinc, mg	11.5 ± 3.3	11.7 ± 3.6	−0.1 ± 2.9	9.6 ± 5.4	7.9 ± 3.1	1.7 ± 5.2 *	10.5 ± 4.7	9.6 ± 3.8	0.9 ± 4.4 *
Copper, mg	2.0 ± 0.5	1.6 ± 0.5	0.5 ± 0.3 *	1.5 ± 0.4	1.1 ± 0.3	0.5 ± 0.3 *	1.8 ± 0.5	1.3 ± 0.5	0.5 ± 0.3
Potassium, mg	3182.9 ± 686.3	3067.8 ± 716.3	115.1 ± 595.9	2503.8 ± 651.8	2416.2 ± 633.5	87.6 ± 397.5	2798.1 ± 744.5	2698.6 ± 741.6	99.5 ± 490.5

* *p* value < 0.05.

**Table 4:** Displacement of macronutrient content of foods after 6 months of walnut supplementation. All values are means of reported intake on 7 24-h recalls.

	Walnut Supplement	Diet		Food Displacement		
Food Groups, Servings per Day	(S) *	Habitual (H)	Walnut (W)	Absolute (D)	%	
				(S + H) − W	D/S X100 **	SEM
Grains, breads, starches	1.24	3.77	3.39	1.62	128.18	9.27
Vegetables and legumes	1.23	3.31	3.12	1.42	112.48	10.82
Fruits and related products	1.20	2.19	2.12	1.27	104.24	8.72
Meat, fish, & poultry	1.17	1.72	1.47	1.42	116.00	6.53
Dairy products and eggs	1.23	3.52	3.34	1.41	107.78	17.34
Fats and oils	1.23	1.93	1.77	1.39	112.78	7.11
Nuts other than walnuts & seeds	1.30	0.82	0.71	1.41	115.44	6.22
All nuts and seeds	1.01	0.75	1.32	0.44	35.10	4.81
Water	1.18	4.91	4.62	1.47	118.49	14.42
Alcoholic beverages	1.32	1.18	1.25	1.25	101.50	6.65
Non-alcoholic beverages	1.20	2.99	3.12	1.07	83.89	10.92
Desserts	1.25	1.19	1.42	1.02	82.80	11.46
Candy, sugar, and sweets	1.23	3.36	3.35	1.24	96.20	24.22
Gravy, soups, and sauces	1.18	1.09	0.98	1.29	100.88	12.10
Condiments	1.23	4.20	4.06	1.37	110.74	18.57

* “S” represents the average intake of the walnut supplement in serving. ** No displacement (0%); partial displacement is >0% to < 100%; full displacement = 100%; more than fully displaced >100%; negative displacement is any % with “−” sign.

**Table 5:** Displacement of specific macronutrients after 6 months of walnut supplementation. All values are means of reported intake on 7 24-h recalls.

	Walnut Supplement	Diet		Nutrient Displacement		
Nutrient	(S) *	Habitual (H)	Walnut (W)	Absolute (D)	%	
				S + H − W	D/S X100 **	SEM
Macronutrients						
Energy, kcal	213.9	1804.0	1914.1	103.8	48.5	7.3
Total Carbohydrate, g	2.3	242.8	238.0	7.1	308.7	18.5
Total Protein, g	3.1	69.7	70.3	2.5	80.6	9.5
Vegetable Protein, g	4.5	26.2	28.6	2.1	46.7	7.2
Total Fat, g	39.1	63.5	79.8	22.8	58.3	8.0
Total SFA, g	2.5	20.8	21.4	1.9	76.0	9.2
Total MUFA, g	1.7	24.1	24.5	1.3	76.5	9.2
MUFA 18:1, g	1.9	22.6	23.0	1.5	78.9	9.4
Total PUFA, g	34.1	13.5	28.1	19.5	57.2	8.0
PUFA 18:2, g	2.64	11.8	23.5	−9.06	−343.2	19.2
PUFA 18:3, g	6.5	1.3	4.4	3.4	52.3	7.6
PUFA 20:4, g	0.3	0.1	0.1	0.3	100.0	10.5
PUFA 20:5, g	0.2	0.1	0.0	0.3	150.0	12.9
PUFA 22:6, g	0.3	0.1	0.1	0.3	100.0	10.5
Alcohol, g	0.5	3.3	2.7	1.1	220.0	15.6
Total Dietary Fiber, g	4.3	19.7	21.1	2.9	67.4	8.7

* “S” average amount of nutrient i contained in the walnut supplement ** No displacement (0%); partial displacement is >0% to < 100%; full displacement = 100%; more than fully displaced >100%; negative displacement is any % with negative (−) sign.

**Table 6:** Displacement of specific micronutrients after 6 months of walnut supplementation. All values are means of reported intake on 7 24-h recalls.

	Walnut Supplement	Diet		Nutrient Displacement		
Nutrient	(S) *	Habitual (H)	Walnut (W)	Absolute (D)	%	
				S + H − W	D/S X100 **	SEM
Vitamins						
Total Vitamin A, µg	91.3	1082.5	1139.1	34.7	38.0	6.5
Vitamin C, mg	0.2	102.9	102.3	0.8	400.0	21.1
Total Folate, µg	18.3	394.2	400.5	12	65.6	8.5
Beta-Carotene, µg	548.4	3878.0	4195.4	231	42.1	6.8
Retinol, µg	7.4	436.2	439.8	3.8	51.4	7.6
Total Vit E, mg	0.1	9.4	9.2	0.3	300.0	18.3
Minerals						
Calcium, mg	80.2	749.7	785.8	44.1	55.0	78.2
Phosphorus, mg	190.3	1149.9	1217.6	122.6	64.4	8.5
Magnesium, mg	70.1	294.1	330.2	34	48.5	7.3
Iron, mg	0.9	14.7	15.1	0.5	55.6	7.9
Zinc, mg	2.1	9.6	10.5	1.2	57.1	8.0
Copper, mg	1.2	1.3	1.8	0.7	58.3	8.1
Potassium, mg	210.4	2698.6	2798.1	110.9	52.7	7.7

* “S” average amount of nutrient i contained in the walnut supplement ** No displacement (0%); partial displacement is >0% to < 100%; full displacement = 100%; more than fully displaced >100%; negative displacement is any % with “−” sign.

## Data Availability

The data presented in this study are available on request from the corresponding author.
